# Poster Session II A328 EVALUATING THE IMPACT OF INCLUDING DIETARY EDUCATION WITHIN AN ELECTRONIC IRRITABLE BOWEL SYNDROME PATHWAY

**DOI:** 10.1093/jcag/gwaf042.328

**Published:** 2026-02-13

**Authors:** N Willett, R Harvie, K Kidd, A Manuel, P Hanias, D Addie, M Stewart, J Jones

**Affiliations:** Division of Digestive Care and Endoscopy, Nova Scotia Health Authority, Halifax, NS, Canada; St Francis Xavier University, Antigonish, NS, Canada; Division of Digestive Care and Endoscopy, Nova Scotia Health Authority, Halifax, NS, Canada; Division of Digestive Care and Endoscopy, Nova Scotia Health Authority, Halifax, NS, Canada; Division of Digestive Care and Endoscopy, Nova Scotia Health Authority, Halifax, NS, Canada; Dalhousie University Faculty of Medicine, Halifax, NS, Canada; Division of Digestive Care and Endoscopy, Nova Scotia Health Authority, Halifax, NS, Canada; Division of Digestive Care and Endoscopy, Nova Scotia Health Authority, Halifax, NS, Canada

## Abstract

**Background:**

Dietary education has been shown to be effective for reducing symptom severity and improving quality of life for patients with IBS. However, not all patients have access to a dietitian through the public health service with skills in managing IBS. There has been a dearth of published literature evaluating the effectiveness of online dietary education courses for IBS patients.

**Aims:**

To determine if the implementation of evidence-based, virtual dietary interventions for IBS is feasible, acceptable, and accessible for Nova Scotians living with IBS, and if it’s more clinically effective and cost effective compared to that provided through a pre-existing, online, group-based education platform.

**Methods:**

This is a hybrid implementation-effectiveness trial comparing the implementation and early effectiveness of two dietary interventions. Participants are randomized to receive either one-on-one virtual dietitian consults (intervention group) or an online group educational program ‘Happy Bellies Nutrition’ for IBS (control group). Data on IBS symptoms and quality of life (QoL) is being collected at baseline and at 5 months. Preliminary analysis is descriptive in nature. This ancillary study has been conducted with Atlantic PATH, under application 2025-101.

**Results:**

A total of 65 participants enrolled, 11 are withdrawn, 24 of those remaining are enrolled in the dietitian arm (Arm A) and 30 are enrolled in the online course arm (Arm B). Of those with baseline data available In Arm A (n = 14) the average age was 55.6 years (sd 7.3) with 78% female (11/14). in Arm B (n = 20) avg age was 54.04 (SD 9.5), also mostly female 90% (18/20)

Both arms began with moderate IBS severity (Arm A mean score (SD): 239.7 (95) Arm B: 272 (78)). Both arms had similar IBS related QoL scores [Arm A: 79.3 (29.1), Arm B: 88 (270)]

Following the interventions, the mean IBS severity score in the dietitian arm was reduced to 167 (89.1). Arm B was reduced to 252 (144). Only the dietitian arm (Arm A) on average, had a reduction to ‘low’ level of IBS severity. In contrast, only Arm B saw an increase in average IBS related quality of life 96.0 (26.9)]. In Arm A, all either somewhat or strongly agreed that they felt confident in managing their IBS at end of study, while in Arm B participants were neutral, somewhat agreed or somewhat disagreed.

**Conclusions:**

Participants are mostly female, with moderate IBS. Early results indicate that there is a reduction in IBS severity after following both interventions. Participants feel more equipped to handle their IBS after speaking with the online dietitian compared to participating in an online course. Findings of this study may justify scaling up dietitian- led IBS treatment services.

**Funding Agencies:**

Research Nova Scotia

